# Social Reminiscence in Older Adults’ Everyday Conversations: Automated Detection Using Natural Language Processing and Machine Learning

**DOI:** 10.2196/19133

**Published:** 2020-09-15

**Authors:** Andrea Ferrario, Burcu Demiray, Kristina Yordanova, Minxia Luo, Mike Martin

**Affiliations:** 1 Department of Management, Technology, and Economics ETH Zurich Zurich Switzerland; 2 Department of Psychology University of Zurich Zurich Switzerland; 3 University Research Priority Program University of Zurich Zurich Switzerland; 4 Collegium Helveticum Zurich Switzerland; 5 Institute of Computer Science University of Rostock Rostock Germany; 6 Institute of Visual & Analytic Computing University of Rostock Rostock Germany; 7 Interdisciplinary Faculty Ageing of Individuals and Society University of Rostock Rostock Germany; 8 School of Psychology, Faculty of Health and Behavioural Sciences University of Queensland Brisbane Australia

**Keywords:** aging, dementia, reminiscence, real-life conversations, electronically activated recorder (EAR), natural language processing, machine learning, imbalanced learning

## Abstract

**Background:**

Reminiscence is the act of thinking or talking about personal experiences that occurred in the past. It is a central task of old age that is essential for healthy aging, and it serves multiple functions, such as decision-making and introspection, transmitting life lessons, and bonding with others. The study of social reminiscence behavior in everyday life can be used to generate data and detect reminiscence from general conversations.

**Objective:**

The aims of this original paper are to (1) preprocess coded transcripts of conversations in German of older adults with natural language processing (NLP), and (2) implement and evaluate learning strategies using different NLP features and machine learning algorithms to detect reminiscence in a corpus of transcripts.

**Methods:**

The methods in this study comprise (1) collecting and coding of transcripts of older adults’ conversations in German, (2) preprocessing transcripts to generate NLP features (bag-of-words models, part-of-speech tags, pretrained German word embeddings), and (3) training machine learning models to detect reminiscence using random forests, support vector machines, and adaptive and extreme gradient boosting algorithms. The data set comprises 2214 transcripts, including 109 transcripts with reminiscence. Due to class imbalance in the data, we introduced three learning strategies: (1) class-weighted learning, (2) a meta-classifier consisting of a voting ensemble, and (3) data augmentation with the Synthetic Minority Oversampling Technique (SMOTE) algorithm. For each learning strategy, we performed cross-validation on a random sample of the training data set of transcripts. We computed the area under the curve (AUC), the average precision (AP), precision, recall, as well as F1 score and specificity measures on the test data, for all combinations of NLP features, algorithms, and learning strategies.

**Results:**

Class-weighted support vector machines on bag-of-words features outperformed all other classifiers (AUC=0.91, AP=0.56, precision=0.5, recall=0.45, F1=0.48, specificity=0.98), followed by support vector machines on SMOTE-augmented data and word embeddings features (AUC=0.89, AP=0.54, precision=0.35, recall=0.59, F1=0.44, specificity=0.94). For the meta-classifier strategy, adaptive and extreme gradient boosting algorithms trained on word embeddings and bag-of-words outperformed all other classifiers and NLP features; however, the performance of the meta-classifier learning strategy was lower compared to other strategies, with highly imbalanced precision-recall trade-offs.

**Conclusions:**

This study provides evidence of the applicability of NLP and machine learning pipelines for the automated detection of reminiscence in older adults’ everyday conversations in German. The methods and findings of this study could be relevant for designing unobtrusive computer systems for the real-time detection of social reminiscence in the everyday life of older adults and classifying their functions. With further improvements, these systems could be deployed in health interventions aimed at improving older adults’ well-being by promoting self-reflection and suggesting coping strategies to be used in the case of dysfunctional reminiscence cases, which can undermine physical and mental health.

## Introduction

### Reminiscence and Healthy Aging

The world’s population is rapidly aging. With its first world report on aging and health [1], the World Health Organization (WHO) promoted a global paradigm shift in aging research by moving from a disease-focused model to a dynamic, contextualized, person-focused model of “healthy aging” [1]. This model emphasizes the interplay of personal characteristics (eg, abilities), environments, and their interactions in producing functioning. Activities represent the interaction between person characteristics and environments; they are understudied in traditional aging research. The novel WHO model encourages aging researchers to step outside the lab and into the real world to examine activities in everyday life [2], aiming to empower individuals to observe, measure, and take earlier action for their own health [3,4]. In this study, we embrace the healthy aging model by examining one such real-life activity: reminiscence.

Reminiscence [5] is the “naturally occurring act of thinking about or telling others about personally meaningful past experiences” [6,7]. These experiences may refer to specific events (eg, the first kiss), repeated ones (eg, going to the gym every Friday), extended ones (eg, a Christmas trip), or even long periods of life (eg, living in a foreign country for some years) [8]. Recalling or sharing valuable life experiences with third parties can support decision-making, bonding with others, and self-understanding [9]. Reminiscing can be a volitional or nonvolitional process recollecting memories [7], an activity that may be private or involve others [7]; in the latter case, we refer to social reminiscence. Many disciplines are interested in the study of reminiscence, such as nursing, social work, education, theology, psychology, and gerontology [10], with a strong focus on reminiscence in the context of aging. Researchers who study aging emphasize a cognitive activity in old age such as reminiscence to be an essential part of healthy aging [11]; in fact, the use of memory interventions and reminiscence in therapies for older adults is common, emphasizing the relation between self-positive functions of reminiscence and well-being [12], according to Webster and Cappeliez’s tripartite model of reminiscence [13].

### Naturalistic Observation as a New Approach to the Study of Reminiscence

The study of reminiscence in older adults has traditionally focused on (1) reflective self-reporting and life reviews [11] and (2) automated reminiscence therapy, that is, “a nonpharmacological intervention involving the prompting of past memories, […] for therapeutic benefits, such as the facilitation of social interactions or the increase of self-esteem” [14], especially for dementia patients.

The use of self-reporting has potential limitations, such as recall biases, response styles, demand characteristics, social desirability, and limitations to introspection [15]. Moreover, the self-report method provides researchers only with the average frequency of an activity over a certain period of time [16]. Studies with a focus on automated reminiscence therapy, in contrast, typically aim at eliciting reminiscence from users (eg, with the remote assistance of a therapist), rather than collecting spontaneous reminiscence events during everyday life settings [17-19].

In 2017, Demiray et al [6] were the first to examine reminiscence using a naturalistic observation method to enable investigating reminiscence in the real world. As opposed to those used in the reminiscence therapies, this method does not rely on self-report and tracks objective behaviors, such as speech in everyday life, with no elicitation of reminiscence events. It can also involve older adults in scientific investigations who would otherwise be excluded from real-life studies relying on self-reporting (eg, older adults who are intimidated by technology or therapy are unable to use a smartphone to complete surveys or to self-report due to worsened eyesight, and/or are part of a clinical population). Furthermore, this method allows for microlongitudinal study designs with many measurement points per participant and both within- and between-persons perspectives in analyses.

However, to understand what kinds of reminiscence patterns are predictive of maintaining healthy aging and quality of life [2], each instance of reminiscing has to be reliably detected in everyday life contexts. In fact, reminiscing is a context-dependent, real-world cognitive activity [20]; if it can be reliably and accurately assessed, it is possible to study the effects of real-world cognitive activities on health outcomes, such as cognitive abilities or cognitive impairments [6,12]. A rich body of literature exists on the link between reminiscence therapies and cognitive and well-being benefits in aging populations: a recent review [21] based on 22 randomized controlled studies showed evidence for the positive effects of reminiscence therapy on quality of life, cognition, and communication in dementia patients. Subramaniam and Woods [22] specifically reviewed the effects of individual-based therapy and concluded that it shows immediate benefits on well-being and cognition. The challenge, however, is to find automated ways to extract and disambiguate the cognitive activity information from data streams collected from many persons in real-world situations [23]. Tracking, detecting, and prompting functional as well as positive reminiscence behaviors in everyday life should allow researchers to design digital interventions to enhance the quality of life of healthy older adults and other patient populations. To these ends, natural language processing (NLP) and machine learning methodologies allow researchers to explore the possibility of reliably predicting reminiscence in combination with naturalistic observation methods and in real time.

### Using NLP and Machine Learning for Reminiscence

Yordanova et al [24] were the first to investigate the applicability of NLP and machine learning methodologies on data from a naturalistic observation study by Demiray et al [6]; they introduced an NLP pipeline and machine learning routines to automatically code the social behaviors and interactions (eg, talking to a partner or daughter/son, giving advice, receive support, etc) in the transcripts of recorded conversations. As coding is a manual process that involves much effort and time, their results showed that the use of NLP and machine learning automation on transcripts of recorded conversations enabled reliable coding of social behaviors and interactions, reducing effort and time. However, they did not consider detecting reminiscence. Their proposed pipeline included data augmentation procedure to cope with highly imbalanced classes, feature engineering based on linguistic, contextual and statistical approaches, and supervised learning with classifiers such as decision trees, random forests (RF), and support vector machines (SVM). As the extreme imbalance between classes poses a central problem in automated coding of textual data, other works propose training classifiers with annotated data sets and later performing manual evaluation to correct misclassified instances by experts [25]. This second manual step is expensive and time-consuming, but it shows that NLP and machine learning deliver promising results with respect to the automated analysis of textual data in social science applications. In fact, the use of NLP and machine learning methods has shown great potential for analyzing social media posts for social affect and behavior, identifying trends in society and demographics, as well as generating predictions of society-changing events, such as diseases [26-28].

Motivated by the availability of reminiscence data from the naturalistic observation study [6] and the results of Yordanova et al [24], this study aims to develop pipelines with NLP and machine learning strategies to automatically detect reminiscence in older adults’ everyday conversations in German using their written transcripts. To do so, we introduce various NLP features (bag-of-words models, part-of-speech [POS] tagging, and pretrained word embeddings) to preprocess written transcripts, which are fed into four families of machine learning algorithms (RF, adaptive boosting [ADA], extreme gradient boosting [XGB], and SVM); multiple learning strategies are setup to cope with class imbalance in data. The methods of this study support the understanding of a key activity of the healthy aging model, that is, reminiscence, in a real-world setting by leveraging transcriptions of everyday life conversations. Moreover, they could support the design of computer systems to detect social reminiscence in the everyday lives of older adults in real time and classify different reminiscence functions. These systems lie at the core of digital health intervention programs [29] aimed at improving older adults’ well-being by promoting self-reflection, as suggested by the healthy aging model. They also provide users with coping strategies for dysfunctional reminiscence [13], which has a negative emotional valence and affects physical and mental health [30-32]. Even when the real-time recording of older adults’ memory activities contained in daily conversations in a research study is shown to be feasible, there are multiple challenges to scaling up this intervention to a large population. However, current findings suggest that despite potential concerns about privacy and data protection issues, there are now a number of technical [33] and analytical solutions for privacy-preserving machine learning for such data [34]. Additionally, a majority of older adults is willing to share portable data collections with researchers [3].

## Methods

### Overview of the Study Design

This study comprises the following steps: (1) collecting data from a naturalistic observation study [6], (2) preprocessing data with NLP methodologies, and (3) training and validating machine learning models to detect reminiscence in a given corpus of transcriptions, by implementing three distinct learning strategies to cope with class imbalance in the data.

### Data Collection: Older Adults’ Everyday Conversations and Reminiscence

#### Data Source

The aim of the naturalistic observation study by Demiray et al [6] was to collect everyday conversations of older adults and examine social reminiscence behavior. Random snippets of older adults’ everyday conversations were collected using the Electronically Activated Recorder (EAR) [35]. They generated 13,275 audio files from 48 older adults (22 men and 26 women) residing in Zurich, Switzerland, over a period of 4 days. The average age of the sample was 70.54 years (SD 4.65, range 62-83 years). To be eligible for the study, participants were required to have a minimum score of 27 on the Mini Mental State Examination [36]. The participants had an average of 10.5 years of education (SD 3.0, range 7-25 years), and they all spoke Swiss German. The study included an introductory session, an observation period, and a feedback session. In the introductory session of the study, participants signed informed consent and received an iPhone in which the EAR was installed. They were informed that the EAR would randomly record a few seconds of audio multiple times per day, except for an automatically inactivated period from every midnight to 6 AM the next day, during the whole observation period. They were told to carry the iPhone and continue daily living, and they were informed that they would not notice when the EAR was recording.

The observation period started the day after the introductory session and lasted 4 consecutive days, during which the audio file recording occurred. After the observation period, the participants were invited to the feedback session, where they returned the phones, completed further questionnaires, and provided their feedback about their experiences of carrying the EAR. They received password-protected CDs containing all of their audio files. All study procedures were approved by the Ethics Research Institute of the Department of Philosophy at the University of Zurich.

#### The EAR App

The EAR (version 2.3.0) [35,37] was installed on each iPhone. It was set to randomly record 30-second audio files 72 times over 4 days. Thus, each participant was recorded 288 times and for a total 144 minutes each. Each iPhone was set to “airplane mode” and locked with a screen-lock password. The participants were instructed to charge the iPhone in the evenings. At the end of the study, the participants reported that the EAR did not affect their daily activities or way of speaking [20].

#### Data Generation: Transcribing and Coding Audio Files

For each audio file, all utterances by the study participants were transcribed by two research assistants, who were fluent in Swiss German and standard written German. Swiss German is an Alemannic dialect spoken in the German-speaking part of Switzerland, which does not have a standard written form. Thus, the Swiss-German dialect in the audio files was translated word-by-word into standard written German and then transcribed. Coders generated binary variables (with values 0 and 1), indicating whether the participant was talking or not and whether he or she was reminiscing or not. The function(s) of reminiscence [8] and participants’ conversation partners (ie, partner/spouse, daughter/son, other family members, etc) were also coded.

#### Coding Reminiscence

Two coders performed the manual coding of reminiscence in each audio file independently. The interrater reliability for the coding of reminiscence, that is, the percentage of audio files with the same coding assigned by both coders, was 95%. All discrepancies in coding were solved by relistening and recoding through discussion. To classify reminiscence, the coders generated a binary variable with values 0 and 1, which correspond to a general conversation and a reminiscence case, respectively.

### Preparing Data

Of the 13,275 audio files generated during the study by Demiray et al [6], 2214 contained conversations of older adults. The data set used for this study comprises these 2214 transcripts, of which 109 (4.9%) were coded as reminiscence. Before applying NLP to the transcripts, we preprocessed the data with regular expressions to remove coding artifacts like “xxx,” “YYY,” “xxxx,” etc, denoting utterances from the audio recording that were not possible to transcribe as well as leading and trailing whitespaces.

### Natural Language Processing of Transcripts

To use written transcripts as inputs of machine learning models, we preprocessed them by computing the NLP features (1) “bag-of-” models on both words and POS tags and (2) real-valued embeddings for each transcript in the data set using pretrained German word embeddings.

#### Bag-of-Words Models

Bag-of-words models [38,39] represent transcripts as real-valued vectors by tokenizing all transcripts in the provided data set, collecting unique tokens, and counting their occurrences before applying normalization (eg, term frequency–inverse document frequency [tf–idf] [38,39]). Bag-of-words models do not consider the order of words in transcripts and generate high-dimensional representation of textual data. In fact, bag-of-words models represent each transcript by a real-valued vector whose dimension is equal to the size of the vocabulary of the whole data set of transcripts. They are widely used for text classification tasks, including studies in digital health [29,40-42]. We computed bag-of-words models using the TfIdfVectorizer() function in the Python sklearn library [43].

#### Bag-of-POS Models

POS tagging is the process of assigning a POS tag to each word in a given corpus [38,39]; the algorithm that performs the tagging is called a POS tagger; a set of all tags is called a tagset. POS tagging enables including information from a word’s context (ie, its relationships with close and related words in a document) in text classification tasks [29]. In this study, we used the POS tagger provided in the core model for the German language “de_core_news_sm,” which is available in the Python library SpaCy [44], to generate the POS tags for all tokens retrieved from the corpus of 2214 transcripts. The SpaCy POS tagger has a tagset comprising 17 distinct tags; similar to bag-of-words models, a bag-of-POS model extracts all POS tags (instead of words) from a transcript and counts their occurrences before applying normalization, such as tf–idf. With bag-of-POS models, one can encode information on the linguistic structure of each transcript in a real-valued, low-dimension representation.

#### Word Embeddings

Word embeddings are real-valued representations of textual data encoding the “distributional hypothesis” [45] about language and words: words that occur in similar contexts tend to be closer to each other as real-valued vectors. Moreover, word embeddings are generally real-vector representations of textual data of much lower dimension than, for example, those in bag-of-words models. They have emerged as a common technique to compute representations of textual data, including studies in digital health [29,40]. In this study, given the limited number of available transcripts, we opted for pretrained German word embeddings using the SpaCy core model for the German language “de_core_news_sm.” The model is “German multi-task CNN trained on the TIGER and WikiNER corpus” [46] and each word embedding has 300 dimensions. The TIGER corpus [47] is curated by the Institute for Natural Language Processing at the University of Stuttgart; it comprises 900,000 tokens from sentences of German text, taken from the Frankfurter Rundschau newspaper [48]. On the other hand, WikiNER [49] is a corpus for multilingual named entity recognition from Wikipedia. In this study, the embedding of each transcript is a real vector of 300 dimensions resulting from averaging the embeddings of all words generated from the given transcript after its tokenization.

### Machine Learning on Transcripts: Learning Strategies

#### Class Imbalance

The data set of 2214 transcripts has 109 records coded as reminiscence; therefore, it has a class imbalance with a ratio of 20:1. A class imbalance [50-53] is a common phenomenon in machine learning and, in particular, in textual data [24,40,54,55]. Since most machine learning algorithms are biased toward the majority class [53], researchers have proposed various solutions to cope with learning with imbalanced data sets [50,51], such as class-based weighting of misclassification errors or data resampling techniques aimed at reducing imbalance [53]. Research has also promoted the use of performance measures, which consider the presence of class imbalance [53] to evaluate machine learning models.

#### Machine Learning Methods

In this study, to cope with class imbalance during the training of machine learning models to detect reminiscence, we implemented the following learning strategies: (1) class-weighted learning (CWL), (2) meta-classifier (MC) learning, and (3) learning in the presence of data augmentation with the Synthetic Minority Oversampling Technique (SMOTE) algorithm [56]. For all the learning strategies, we trained machine learning classifiers [57] using the RF, ADA, XGB [58], and SVM algorithms fed on all NLP features: bag-of-words, bag-of-POS NLP models, and pretrained word embeddings. In the cases of RF or boosting algorithms trained on either bag-of-words or bag-of-POS features, we computed feature importance using the sklearn property .feature_importances_. Table 1 summarizes all the NLP features, classifiers, and learning strategies used in this study.

**Table 1:** Summary of all natural language processing (NLP) features, machine learning algorithms (ie, classifiers), and learning strategies considered in this study.

Features, algorithms, and strategies	Cases
NLP features	Bag-of-words Bag-of-POS^a^ Pretrained German word embeddings
Algorithm (classifier)	Random forests Adaptive boosting Extreme gradient boosting Support vector machines
Learning strategy	Class-weighted training Meta-classifier training Data augmentation with SMOTE^b^

^a^POS: part-of-speech.

^b^SMOTE: Synthetic Minority Oversampling Technique.

#### Evaluation Metrics

For all the learning strategies, the performance of the machine learning models on test data was computed using the area under curve (AUC), precision, recall (or sensitivity), the average precision (AP), specificity, and the F1 score measures. The AUC summarizes in a single number the performance of the classifier shown in the receiver operating curve [59,60], which plots the true positive rate versus the false positive rate, at various classifier thresholds settings. Precision is the number of true positives (ie, transcripts containing reminiscence, which are correctly predicted by the machine learning model) divided by the number of transcripts, which are predicted to contain reminiscence by the model. Recall (or sensitivity) is the number of true positives divided by the number of transcripts containing reminiscence. The sklearn implementation of the AP [61] summarizes the precision-recall curve [62] as the weighted mean of precisions achieved at each threshold by the classifier, the increase in recall from the previous threshold used as the weighting. We chose to report the AUC and AP, as they provide a global overview of the classifier performance, for all possible classification thresholds. Specificity is the number of true negatives divided by the total number of negative instances, while the F1 score is the harmonic mean of precision and recall measures [38]; it is a common evaluation metric in the presence of imbalanced data.

The formulas for AP and F1 score are as follows:


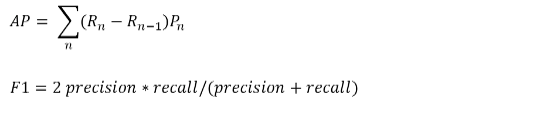


In the AP formula, *R_n_* denotes the recall computed at step *n* (similarly for *R_n_*_–1_), while *P_n_* is the precision at step *n*.

#### Error Analysis

To identify errors in detecting reminiscence, we analyzed the false positives with the highest predicted probabilities (10% of all cases) and the false negatives with the lowest predicted probabilities (10% of all cases) computed for all the models presented in Table 2.

#### Experimental Setting

##### Class-Weighted Learning

**Figure 1:**
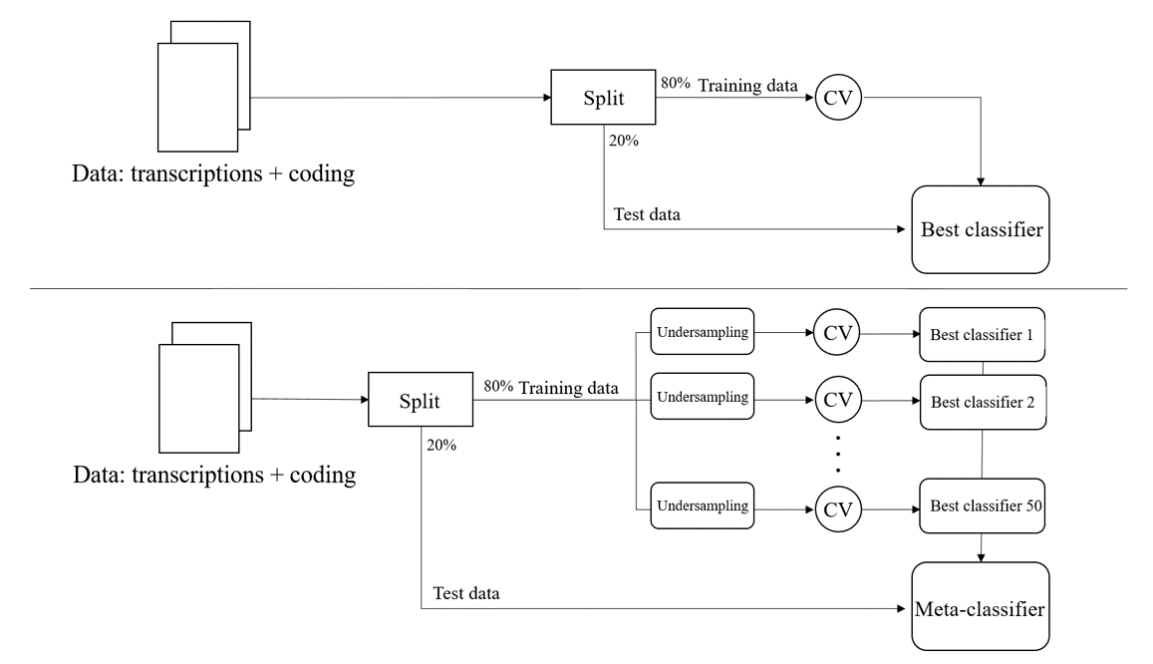
Top panel: Class-weighted learning and data augmentation learning strategies. We performed a single data partition into train and test sets; the best classifier emerged from 5-fold cross-validation (CV). Bottom panel: meta-classifier learning strategy; we undersampled the training data 50 times, collecting 50 distinct models in a voting ensemble after CV. We applied the three strategies for all the combinations of natural language processing features and machine learning algorithms shown in Table 1.

We show the CWL strategy in Figure 1, top panel. After an initial partition of the data into train and test subsets with a 80:20 ratio, a 5-fold cross-validation routine was applied to the 1771 training data (87 reminiscence) to select the best model for all NLP features and families of classifiers shown in Table 1. We use the AUC to measure performance on the validation folds. The CWL strategy does not modify the imbalanced class distribution of training data, but reweighs them according to their class during the training of the machine learning algorithm at hand [40,52,54], penalizing the cost of misclassifying data points from the minority class [52,63,64]. For example, for the RF, ADA, and SVM algorithms, we selected the parameter “*class_weight=balanced*” [65] in their sklearn implementations. Similar considerations held for XGB algorithms, where we selected “*scale_pos_weight=weight*,” where *weight* denoted the ratio of the number of negative class samples to the positive class (ie, reminiscence) [66]. The best model resulting from the cross-validation was evaluated on test data, by computing AUC, AP, and F1.

##### Meta-Classifier Training

We show the MC strategy in Figure 1 (bottom panel). The MC is a majority voting ensemble classifier [67] comprising 50 equally weighted distinct models resulting from 50 runs of 5-fold cross-validation on 50 randomly undersampled (with 1:1 ratio) training data sets. Undersampling is a common technique in imbalanced learning [41,68]; the use of voting allows reducing the bias of a single undersampling of training data. For each run, the cross-validation routine was performed on 174 training transcripts (87 reminiscence). The AUC and AP of the MC were computed on test data.

##### Data Augmentation With SMOTE

Figure 1 (top panel) shows the strategy involving data augmentation with the SMOTE algorithm: it follows the same steps as the one implemented for CWL. However, during the 5-fold cross-validation, the results of all NLP pipelines on transcripts are preprocessed with SMOTE before being fed into the machine learning classifiers. SMOTE [50] is an algorithm that generates synthetic examples of the minority class (in this study, the reminiscence class) in imbalanced data sets; given a minority class data point, SMOTE generates synthetic examples along line segments connecting the given data point to its K nearest neighbors (K=5 is the default value). SMOTE is widely used to perform data augmentation in the presence of imbalanced learning, including clinical studies [40,69,70]. The Python implementation of the SMOTE algorithm also allows controlling the oversampling quota, that is, the ratio between the minority class (after resampling) and the majority class. For example, using SMOTE on training data to reach a 1:1 ratio among classes, we ran the cross-validation routines in Figure 1 (top panel) on a total of 3368 data points (1684 reminiscence).

##### Cross-Validation

For all three learning strategies, as shown in Figure 1, we performed cross-validation by tuning the hyperparameters of (1) the NLP pipelines computing bag-of-words and bag-of-POS features, together with those of (2) the machine learning algorithms before retrieving the best classifier (MC in the case of the voting learning strategy).

##### NLP Preprocessing Pipelines

For the bag-of-words features, we tuned the n-grams, German stopword list (ie, its removal or not), minimum document frequency, maximum document frequency, and maximum number of feature hyperparameters. After preprocessing, in case of 1-grams we have 6596 tokens, and 38,347 in the case of 2-grams. The preprocessing of the POS features follows the one for bag-of-words, with the exception of the German stopword list; in case of 1-grams we obtained 16 tokens, and 270 in the case of 2-grams.

##### Machine Learning Models: Hyperparameters

For ADA classifiers, we tuned the number of estimators and the learning rate; for XGB, we tuned the number of estimators, the learning rate, and the maximum tree depth; and for RF, we tuned the number of trees in the ensemble, their depth, and the number of features considered at each tree split during training. In addition, when considering the data augmentation learning strategy, we tuned the SMOTE number of nearest neighbors and oversampling quota hyperparameters. The NLP preprocessing pipelines are the same for all machine learning models.

## Results

### Machine Learning Modeling

Multimedia Appendix 1 displays the results of the machine learning modeling, reporting the best performing classifier for all NLP features and learning strategies, together with its AUC, AP, precision, recall, F1, and specificity performance measures, which we computed on test data (443 transcripts, 22 reminiscence).

Table 2 displays the two best performing classifiers (considering the F1 score as performance measure) for each learning strategy. We choose two best models per learning strategy to show how different combinations of machine learning algorithms and NLP features may result in different precision-recall trade-offs.

Considering the class weighting strategy, SVM outperforms all other classifiers, with the highest F1 score (0.48) when trained on bag-of-words features, with a balanced precision-recall trade-off and very high specificity (0.98). On the other hand, SVM trained on word embeddings shows higher AP (0.61) and recall (0.77); however, its lower precision (0.21) results in a lower F1 score (0.33), together with a lower specificity (0.85).

The best performing classifiers for the MC strategy, which are trained on word embeddings and bag-of-words features, show F1 scores (0.30 for both ADA and XGB classifiers) lower than those in the class-weighted and data augmentation strategies. This is due to low precision; similarly, they show low specificities. However, they reach very high recall (1.00 for ADA and 0.86 for XGB classifiers) and high AUCs.

Finally, for the data augmentation strategy, SVM on word embeddings shows highest F1 score (0.44), with recall (0.59) higher than precision (0.35) and high specificity (0.94). On the other hand, ADA classifiers trained on word embeddings show a more balanced precision-recall trade-off, with a higher precision (0.43) but lower recall (0.41) than the SVM classifier, resulting in a slightly lower F1 score (0.42) but higher specificity (0.97).

**Table 2:** Summary of best models for each learning strategy, considering the F1 score.

Learning strategy and NLP^a^ feature			Classifier family		AUC^b^		AP^c^		Precision		Recall		F1		Specificity
**Class weighting**															
	BOW^d^	SVM^e^		0.91		0.56		0.50		0.45		0.48		0.98	
	EMB^f^	SVM		0.91		0.61		0.21		0.77		0.33		0.85	
**Meta-classifier**															
	BOW	XGB^g^		0.90		0.45		0.18		0.86		0.30		0.79	
	EMB	ADA^h^		0.92		0.38		0.18		1.00		0.30		0.76	
**Data augmentation**															
	EMB	SVM		0.89		0.54		0.35		0.59		0.44		0.94	
	EMB	ADA		0.84		0.36		0.43		0.41		0.42		0.97	

^a^NLP: natural language processing.

^b^AUC: area under the curve.

^c^AP: average precision.

^d^BOW: bag-of-words.

^e^SVM: support vector machines.

^f^EMB: word embeddings.

^g^XGB: extreme gradient boosting.

^h^ADA: adaptive boosting.

### Feature Analysis

Overall, considering Table 2 and the F1 score, the use of word embeddings outperformed other NLP features for the data augmentation strategy; it delivers performance equal to the one with bag-of-words features for the MC strategy; on the other hand, for class weighting, bag-of-words features deliver the highest performance with the SVM classifier.

Considering bag-of-words features, for the data augmentation strategy XGB outperformed the other classifiers (with 1-grams, and no German stopword removal) with 300 boosting iterations, shallow trees (ie, a depth of 1), and the number of neighbors used by SMOTE equal to K=13. The words with the highest feature importance were German stopwords (eg, “gewesen,” “und,” “wir,” “ist,” and “ich” [“been,” “and,” “we,” “is,” and “I”]). Training the same classifier but removing the stopwords led to a strong decline in performance (AUC=0.79, AP=0.22, F1 = 0.27); in this case, the words with the highest feature importance comprised adverbs (eg, “aber,” and “einfach” [“but” and “simply”]) and past participles (eg, “gesagt” and “gehabt” [“said” and “had”]), among others.

Considering POS features and the class weighting strategy, XGB delivered the highest performance in the presence of 1- and 2-grams, with few boosting iterations (ie, 50) and shallow trees (ie, a depth of 1). The 1-grams with the highest feature importance were auxiliary verb forms (eg, imperative, infinitive, perfect participle of “sein,” “haben,” and “werden” [ie, “to be,” “to have,” and “to become”]), conjunctions, and adverbs, while the 2-grams comprised verbs and auxiliary verbs (eg, the combinations of a noun and the past participle of “sein,” “haben,” and “werden”), adverbs and verbs (eg, “aber weisst,” “dann sagt,” or “mehr gegessen”).

On the other hand, considering the data augmentation strategy and POS features, the XGB algorithm presented in Multimedia Appendix 1 performs on 10 boosting iterations, in the presence of shallow trees, 2-grams, and K=5 neighbors used by SMOTE. In the case of the class weighted learning strategy and POS features, it performs on 50 boosting iterations, shallow trees, and 2-grams.

The POS 2-grams with the highest feature importance comprised punctuation signs followed by a conjunction (eg, “. Und,” “. Aber,” or “, oder”), adverbs and auxiliary verbs (eg, “dann habe/n” and “da hat/ben”), prepositions and determinative articles (eg, “mit einer/m” and “mit der/m,” [ie, “with a(n)” and “with the”]) and adverbs followed by verbs (eg, “aber weisst,” “mehr gegessen,” and “selber gefahren”).

Finally, considering word embeddings, SVM with radial basis kernel (and scaling) on augmented data outperformed all other classifiers, with K=5 neighbors used by SMOTE (F1=0.44), followed by ADA algorithms (F1=0.42) on K=9 SMOTE neighbors. The model with SVM shows much higher recall (0.59), AUC, and AP, while the model with ADA improves precision (0.43) and consequently specificity (0.97).

## Discussion

### Principal Results

The primary purpose of this study was to leverage NLP features and machine learning strategies to detect reminiscence in the conversations of older adults in German in a naturalistic observation study. We used the written transcripts of the conversations and a manually coded variable (reminiscence or not reminiscence) as the basis for the prediction. We considered a wide array of methodologies, including different NLP features (ie, bag-of-words, POS tagging, and pretrained German word embeddings), multiple machine learning algorithms, and learning strategies to handle class imbalance. Results indicate that selected combinations (see below) of learning strategies, NLP features, and machine learning models show the potential to detect reminiscence. We argue that their performance can be further improved through feature engineering, by combining NLP features, using NLP-driven data augmentation techniques and collecting more data.

#### Learning Strategies

Class weighting SVM outperforms others machine learning models, with the highest performance seen when trained on bag-of-words (F1=0.48) with a balanced precision-recall trade-off, high specificity (0.98), AUC=0.91, and AP=0.56. MC strategies show lower F1 scores than class weighted and data augmentation ones, with highly imbalanced precision-recall trade-offs, resulting in low precision and, consequently, low F1 score and specificity. On the other hand, data augmentation with SMOTE delivers performance comparable to the one of class weighting, although only when using word embeddings. However, SMOTE is a purely computational approach to data augmentation, as it generates new data points from any numerical representation of transcripts (eg, on word embeddings). On the other hand, data augmentation algorithms, such as replacing with synonyms, random inserting, swapping, or deleting words [71], process transcripts directly and have improved text classification performance across multiple data sets [71]. We will further investigate the use of NLP-driven data augmentation methodologies and feature engineering [72] in forthcoming studies.

#### Machine Learning Classifiers

Overall, SVM delivered the highest performance across the class-weighted and data augmentation learning strategies, and boosting methodologies for the MC strategy. SVM proved to exhibit competitive performance in NLP tasks with imbalanced data sets, such as detecting offensive language (including German) [55,73,74], while the XGB algorithm proved its effectiveness in a vast array of machine learning problems [75,76]. Consequently, RF learning methods were outperformed by all learning strategies and NLP features.

#### NLP Features

Bag-of-words and word embeddings delivered the highest performance of all learning strategies; machine learning models trained on POS features delivered low performance due to their low precision (combined with low recall, such as in the case of ADA for class weighting and data augmentation strategies). Considering models trained on bag-of-words features, German stopwords related to past tense (eg, “gewesen”), personal pronouns (eg, “ich” and “wir”), and connecting words (eg, “und” and “aber”) showed high feature importance, suggesting that they can encode the time, personal narrative, and structure of reminiscence. Models trained on POS tags, however, use both 1-gram and 2-grams; in particular, 2-grams comprising punctuation signs followed by a conjunction, such as “. Und” and “. Aber,” suggest that reminiscing is a multisentence act during which the speaker pauses and uses forms of emphasis to elevate certain clauses to positions of more influence and importance. We will further investigate these points in future studies, introducing linguistic measures for reminiscence transcripts like other studies [24,77] to improve bag-of-words and POS models with additional sets of features to encode the phrase structure of a sentence and combining bag-of-words and POS features together.

#### Error Analysis

Based on an error analysis conducted on the false positives and false negatives for all the models in Table 2, the models tend to predict long transcripts with multiple sentences referring to the past incorrectly as reminiscence (ie, false positives). This results in low precision, affecting the F1 score of all classifiers. On the other hand, false negatives (ie, transcripts incorrectly classified as not reminiscence) are typically short transcripts with few words; more coded transcriptions will help to reduce both errors, in particular the false positives, enlarging the corpus of conversations and improving vocabulary richness.

In this study, we considered only transcripts in German; therefore, we have no specific insight into the specific challenges of detecting reminiscence in another language, such as English. However, earlier studies [24] have compared the application of NLP methods on transcripts of daily conversations in both German and English. Evaluated on the same set of methods and analysis pipeline, the results have suggested no significant difference in performance between the two languages. The use of different NLP features, classifiers, and learning strategies discussed in this study seems promising to develop a system for the real-time detection of reminiscence in everyday conversations in German of older adults. Such a system could leverage audio-to-text software [78] of advanced methods from automated coding [24] to automate the transcription of conversations before NLP preprocessing and the computation of machine learning predictions.

### Limitations

This study has several limitations. The data set of conversations used to classify reminiscence had a limited number of records due to the short duration of the naturalistic observation study (4 days) [6]. Moreover, as we considered data from a single observation study, we cannot infer the generalizability of the presented results to other data sources. The results are based on a single partitioning of data into train and test sets. In this study, we used SpaCy pretrained German word embeddings; clearly, these embeddings are trained on corpora that may not fully encode the linguistic specificities characterizing conversations among older adults. In addition, due to the small data set available for this study, we refrained from training embeddings as well as performing machine learning with data-intensive deep learning models [79].

### Comparison With Prior Work

Previous research focused on reminiscence as a therapy against dementia have shown the efficacy of information and communication technologies [14]; in particular, studies addressing the topic of automated reminiscence therapy aim at eliciting reminiscence from the users rather than detecting the presence of reminiscence in everyday life [17,18]. These studies have assumed that reminiscence is elicited in a setting with a human companion (eg, a therapist) or through a digital companion device using the Wizard of Oz technique [19]. Few studies have attempted to identify the presence of reminiscence with methods from pattern recognition and machine learning. For example, Naini et al [80] proposed a machine learning model for ranking posts from social media to create life summaries and retain memorable Facebook posts, that is, posts in a user’s timeline worth remembering. A retention model based on the learning-to-rank RankSVM algorithm [81] selects posts. Alternatively, Kikhia et al [82] proposed a method to identify places of importance in lifelogging events using clustering methods and locational sensor data. The use of lifelogs allows individuals to reminisce by recalling memories, experiences, and valuable past events for fun, as a personal diary, or as a support for people with memory problems [83].

Data from Demiray et al’s observational study [6] have been recently used in studies using NLP methodologies and machine learning. For example, one study [24] used their data to address the more general problem of coding social and cognitive variables from the transcripts of older adults’ daily conversations. They trained multiple families of classifiers (ie, SVM, decision trees, and RF) on NLP features, including latent Dirichlet allocations and data augmentation, achieving good performance in different categories. Another study [77] used their data to compute linguistic features (ie, entropy and number of clauses) to examine the effects of age on the use of real-life language in contexts with social interactions. However, to our knowledge, this is the first study investigating the detection of reminiscence directly from the transcripts of daily conversations. Due to the presence of imbalanced data and the type of language (German) used in the conversations, this study addresses problems that are only partially present in prior scientific works in the reminiscence literature.

### Conclusions

This work provides evidence to support the use of NLP and machine learning in detecting reminiscence based on written transcripts of older adults’ everyday conversations in German. These results represent a novelty in the literature on reminiscence. The proposed methodology can be applied to larger natural observation studies to investigate the differences in reminiscing among cultures, countries, and social contexts using, in particular, lexical features and linguistic measures.

The aim of the WHO healthy aging model is to empower individuals to observe, measure, monitor, and take earlier action for their own health; reminiscence is an essential activity for promoting healthy aging among older adults. Real-time detection of reminiscence in everyday conversations can offer valuable information for older adults to understand their own health behaviors in everyday life and to support their autonomy in health maintenance.

Therefore, the training of high-performance classifiers supports the design of digital health interventions to improve older adults’ quality of life by supporting their healthy aging through the real-time monitoring of reminiscence events in everyday conversations. The digital interventions may consist of a daily diary function to support the contextualization of identified reminiscing events and suggest reminiscence-related positive activities, such as replaying positive life stories and prompting more active social interactions. Another possible implementation is a conversational agent–based digital support function to support users in detecting dysfunctional reminiscence events by suggesting coping strategies. The effectiveness of the interventions could be measured by quantifying their effect on depression, quality of life, and social behavior scores, therefore assessing the impact of reminiscence therapies on healthy aging in everyday life contexts.

## Appendix

Multimedia Appendix 1 Machine learning modeling: all results.

## Abbreviations

ADA: adaptive boosting
AP: average precision
AUC: area under the curve
CLW: class-weighted learning
EAR: Electronically Activated Recorder
NLP: natural language processing
POS: part-of-speech
RF: random forests
SMOTE: Synthetic Minority Oversampling Technique
SVM: support vector machines
tf–idf: term frequency–inverse document frequency
WHO: World Health Organization
XGB: extreme gradient boosting
