# Supplementary material for: Social Reminiscence in Older Adults’ Everyday Conversations: Automated Detection Using Natural Language Processing and Machine Learning
Source: J Med Internet Res. 2020 Sep 15;22(9):e19133. doi: 10.2196/19133 (PMC7525396; doi:10.2196/19133)
Supplement: Multimedia Appendix 1 [file jmir_v22i9e19133_app1.docx]

**Machine** **learning modeling: all results.**

We report the best performing classifier for all NLP features and learning strategies, together with its AUC, AP, precision, recall, F1 and specificity computed on test data.

| **Learning Strategy** | **NLP Feature** | **Classifier Family** | **AUC** | **AP** | **Precision** | **Recall** | **F1** | **Specificity** |
| --- | --- | --- | --- | --- | --- | --- | --- | --- |
| Class Weighting | BOW | ADA | 0.63 | 0.11 | 0.11 | 0.14 | 0.12 | 0.94 |
|  | BOW | RF | 0.89 | 0.38 | 0.19 | 0.82 | 0.31 | 0.82 |
|  | BOW | SVM | 0.91 | 0.56 | 0.50 | 0.45 | 0.48 | 0.98 |
|  | BOW | XGB | 0.82 | 0.3 | 0.14 | 0.68 | 0.23 | 0.77 |
|  | EMB | ADA | 0.85 | 0.34 | 0.46 | 0.27 | 0.34 | 0.98 |
|  | EMB | RF | 0.90 | 0.47 | 0.14 | 0.91 | 0.25 | 0.72 |
|  | EMB | SVM | 0.91 | 0.61 | 0.21 | 0.77 | 0.33 | 0.85 |
|  | EMB | XGB | 0.89 | 0.45 | 0.15 | 0.68 | 0.24 | 0.80 |
|  | POS | ADA | 0.73 | 0.17 | 0.26 | 0.27 | 0.27 | 0.96 |
|  | POS | RF | 0.89 | 0.3 | 0.15 | 0.91 | 0.26 | 0.74 |
|  | POS | SVM | 0.84 | 0.31 | 0.14 | 0.77 | 0.24 | 0.75 |
|  | POS | XGB | 0.89 | 0.4 | 0.16 | 0.77 | 0.27 | 0.79 |
| Data Augmentation | BOW | ADA | 0.42 | 0.14 | 0.24 | 0.32 | 0.22 | 0.95 |
|  | BOW | RF | 0.86 | 0.32 | 0.21 | 0.73 | 0.32 | 0.86 |
|  | BOW | SVM | 0.62 | 0.09 | 0.14 | 0.14 | 0.14 | 0.96 |
|  | BOW | XGB | 0.88 | 0.37 | 0.25 | 0.59 | 0.36 | 0.91 |
|  | EMB | ADA | 0.84 | 0.36 | 0.43 | 0.41 | 0.42 | 0.97 |
|  | EMB | RF | 0.91 | 0.54 | 0.15 | 0.91 | 0.26 | 0.73 |
|  | EMB | SVM | 0.89 | 0.54 | 0.35 | 0.59 | 0.44 | 0.94 |
|  | EMB | XGB | 0.89 | 0.38 | 0.21 | 0.77 | 0.33 | 0.85 |
|  | POS | ADA | 0.68 | 0.13 | 0.16 | 0.27 | 0.20 | 0.92 |
|  | POS | RF | 0.83 | 0.17 | 0.14 | 0.77 | 0.23 | 0.75 |
|  | POS | SVM | 0.83 | 0.30 | 0.17 | 0.59 | 0.26 | 0.85 |
|  | POS | XGB | 0.85 | 0.29 | 0.17 | 0.59 | 0.26 | 0.85 |
| Meta-Classifier | BOW | ADA | 0.89 | 0.50 | 0.15 | 0.91 | 0.26 | 0.74 |
|  | BOW | RF | 0.92 | 0.56 | 0.17 | 0.86 | 0.28 | 0.77 |
|  | BOW | SVM | 0.92 | 0.54 | 0.14 | 0.95 | 0.24 | 0.69 |
|  | BOW | XGB | 0.90 | 0.45 | 0.18 | 0.86 | 0.30 | 0.79 |
|  | EMB | ADA | 0.92 | 0.38 | 0.18 | 1.00 | 0.30 | 0.76 |
|  | EMB | RF | 0.93 | 0.50 | 0.17 | 0.95 | 0.28 | 0.75 |
|  | EMB | SVM | 0.92 | 0.54 | 0.13 | 0.95 | 0.23 | 0.68 |
|  | EMB | XGB | 0.93 | 0.58 | 0.16 | 0.95 | 0.27 | 0.73 |
|  | POS | ADA | 0.90 | 0.35 | 0.14 | 0.86 | 0.24 | 0.71 |
|  | POS | RF | 0.92 | 0.43 | 0.16 | 0.86 | 0.27 | 0.76 |
|  | POS | SVM | 0.93 | 0.51 | 0.15 | 0.86 | 0.25 | 0.74 |
|  | POS | XGB | 0.90 | 0.43 | 0.15 | 0.91 | 0.26 | 0.74 |
